# Effects of Sodium-Glucose Cotransporter-2 Inhibitors on Left Ventricular Global Longitudinal Strain in Adults with Type 2 Diabetes Mellitus: A Systematic Review

**DOI:** 10.3390/jcm15135137

**Published:** 2026-07-01

**Authors:** Larissa Dăniluc, Răzvan Dăniluc, Adela Benea, Alexandra-Iulia Lazăr-Höcher, Claudia Raluca Balasa Virzob, Mihaela-Diana Popa, Razvan Susan, Adina Braha, Adrian Apostol, Alexandra Sima, Lina Haj Ali, Loredana Suhov, Delia Hutanu, Mihaela Viviana Ivan

**Affiliations:** 1 Doctoral School, “Victor Babes” University of Medicine and Pharmacy, Eftimie Murgu Sq. No. 2, 300041 Timisoara, Romania; larissa.daniluc@umft.ro (L.D.); razvan.daniluc@umft.ro (R.D.); adela.benea@umft.ro (A.B.); alexandra.hocher@umft.ro (A.-I.L.-H.); lina.haj-ali@umft.ro (L.H.A.); loredana.ogarcin@umft.ro (L.S.); 2 Department of Cardiology, “Pius Brinzeu” Clinical Emergency County Hospital Timisoara, 300736 Timisoara, Romania; ivan.viviana@umft.ro; 3 Discipline of Cardiology, Department VII, Internal Medicine II, “Victor Babes” University of Medicine and Pharmacy, Eftimie Murgu Sq. No. 2, 300041 Timisoara, Romania; 4 Clinical Hospital of Infectious Diseases and Pulmonology “Victor Babes”, Gheorghe Adam Street 13, 300310 Timisoara, Romania; 5 Research Center, Institute of Cardiovascular Diseases Timisoara, 300310 Timisoara, Romania; 6 Department of Clinic Nursing, “Victor Babes” University of Medicine and Pharmacy Timisoara, Eftimie Murgu Square 2, 300041 Timisoara, Romania; virzob.claudia@umft.ro; 7 Department of Microbiology, “Victor Babes” University of Medicine and Pharmacy Timisoara, Eftimie Murgu Square 2, 300041 Timisoara, Romania; popa.mihaela@umft.ro; 8 Department of Family Medicine, Centre for Preventive Medicine, “Victor Babes” University of Medicine and Pharmacy, 300041 Timisoara, Romania; razvansusan@umft.ro; 9 Second Department of Internal Medicine, “Victor Babes” University of Medicine and Pharmacy, 300041 Timisoara, Romania; braha.adina@umft.ro (A.B.); sima.alexandra@umft.ro (A.S.); 10 Center for Molecular Research in Nephrology and Vascular Disease, “Victor Babes” University of Medicine and Pharmacy, 300041 Timisoara, Romania; 11 Biology Department, Chemistry-Biology-Geography Faculty, West University of Timisoara, 300115 Timisoara, Romania; delia.hutanu@e-uvt.ro

**Keywords:** sodium-glucose cotransporter-2 inhibitors, left ventricular global longitudinal strain, type 2 diabetes mellitus, speckle-tracking echocardiography, myocardial deformation

## Abstract

**Background:** Type 2 diabetes mellitus (T2DM) is associated with subclinical myocardial dysfunction, which may occur despite preserved left ventricular ejection fraction. Left ventricular global longitudinal strain (LV GLS) is a sensitive marker of early systolic impairment and may detect subtle changes in myocardial function before conventional echocardiographic parameters become abnormal. The effect of sodium-glucose cotransporter-2 inhibitors (SGLT2i) on LV GLS in adults with T2DM remains incompletely defined. **Objective:** To synthesize the available evidence on the effects of SGLT2i therapy on LV GLS or LV strain in adults with T2DM. **Methods:** Original full-text human studies evaluating SGLT2i therapy in adults with T2DM and reporting LV GLS or LV strain were included. LV GLS was assessed primarily by speckle-tracking echocardiography, while one study used cardiac magnetic resonance feature-tracking. Reviews, conference abstracts, protocols, animal-only studies, and studies without LV strain assessment were excluded. Risk of bias was assessed using RoB 2 for randomized studies and ROBINS-I for non-randomized studies. **Results:** Twenty-six studies involving more than 2300 participants were included. The studies evaluated dapagliflozin, empagliflozin, ertugliflozin, canagliflozin, or mixed SGLT2i regimens across heterogeneous clinical populations, including patients with preserved ejection fraction, pre-heart failure, diabetes-related cardiomyopathy, chronic heart failure, coronary artery disease, hypertension, non-alcoholic fatty liver disease, and cardio-oncology risk. Most observational and before–after studies reported favorable changes in LV GLS after SGLT2i therapy, whereas randomized and controlled studies showed more variable findings. Several studies also reported improvements in LV remodeling, diastolic function, left atrial function, myocardial work indices, NT-proBNP, cardiometabolic parameters, or epicardial adipose tissue thickness. However, the certainty of evidence was limited by methodological heterogeneity, differences in comparator groups, variable follow-up duration, non-standardized imaging protocols, and risk of bias, particularly in non-randomized and single-arm studies. **Conclusions:** SGLT2i therapy may be associated with favorable changes in LV GLS in adults with T2DM, suggesting a potential beneficial effect on subclinical left ventricular systolic function. However, current evidence does not definitively establish a consistent treatment effect across all populations. Larger randomized controlled trials with standardized strain imaging protocols, predefined LV GLS endpoints, and clinically relevant follow-up are needed to determine whether SGLT2i-related improvements in LV GLS reflect true myocardial benefit and translate into improved cardiovascular outcomes.

## 1. Introduction

Type 2 diabetes mellitus (T2DM) is a major cardiometabolic disorder associated with an increased risk of cardiovascular disease, heart failure, chronic kidney disease, disability, and premature [1]. Cardiovascular complications remain a leading cause of morbidity and mortality in adults with T2DM, while heart failure is increasingly recognized as an important diabetes-related complication [1]. The global burden of diabetes continues to increase, emphasizing the need for early identification of cardiovascular involvement, improved risk stratification, and strategies aimed at preventing progression toward overt cardiac dysfunction [2,3].

Left ventricular ejection fraction (LVEF) remains one of the most widely used echocardiographic parameters for assessing systolic function. However, LVEF may remain preserved despite early myocardial injury, particularly in patients with metabolic disease or subclinical cardiac involvement. Left ventricular global longitudinal strain (LV GLS), derived from speckle-tracking echocardiography, provides a more sensitive assessment of myocardial deformation and can detect subtle systolic impairment before conventional measures become abnormal [4,5,6]. For this reason, LV GLS has gained increasing importance as a diagnostic and prognostic marker across different cardiovascular settings [4,5,6].

The role of LV GLS is particularly relevant in T2DM. Patients with T2DM may develop subclinical myocardial dysfunction and diastolic abnormalities even in the absence of overt heart failure and despite preserved LVEF [7,8]. This suggests that diabetes-related myocardial involvement may begin before clinically apparent cardiovascular disease. Early identification of such abnormalities is clinically important because it may help improve cardiovascular risk stratification, guide closer follow-up, and support the evaluation of therapies with potential myocardial benefits.

Diabetes-related myocardial disease is not restricted to mild or clinically silent abnormalities. Depending on disease duration, comorbidities, coronary microvascular function, and loading conditions, T2DM may be associated with clinically relevant systolic and diastolic dysfunction, myocardial fibrosis, impaired relaxation, abnormal ventricular-arterial coupling, and reduced myocardial reserve. Therefore, the term subclinical dysfunction in this review refers primarily to dysfunction detected before overt heart failure or before a reduction in conventional LVEF, not to the absence of biologically meaningful myocardial disease.

Sodium-glucose cotransporter-2 inhibitors (SGLT2i) have become an important component of cardiovascular and cardiorenal risk reduction in adults with T2DM, especially in patients with established cardiovascular disease, heart failure, chronic kidney disease, or multiple cardiovascular risk factors [1]. Large cardiovascular outcome trials have demonstrated important clinical benefits of this drug class, including improved cardiovascular outcomes with empagliflozin in patients with T2DM and high cardiovascular risk and reduced heart failure-related outcomes with dapagliflozin in patients with heart failure with reduced ejection fraction [9,10].

Several mechanisms may contribute to the cardiovascular effects of SGLT2i, including natriuresis, osmotic diuresis, reductions in preload and afterload, improved myocardial energetics, reduced inflammation and oxidative stress, improved endothelial function, and potential effects on myocardial fibrosis and remodeling. However, whether these mechanisms translate into measurable improvement in subclinical myocardial function remains incompletely defined. In this context, LV GLS may be a useful imaging marker for evaluating early myocardial changes associated with SGLT2i therapy.

Preclinical and translational evidence further supports this rationale. Beyond glycosuria and natriuresis, SGLT2 inhibitors may exert direct or indirect effects on cardiomyocytes, endothelial cells, vascular smooth muscle cells, coronary microcirculation, capillarization, nitric oxide signaling, oxidative stress, inflammation, mitochondrial function, myocardial energetics, and fibrosis. These vascular and myocardial mechanisms provide a biologically plausible basis for assessing whether SGLT2i therapy is associated with changes in LV GLS, an imaging marker sensitive to early systolic impairment.

Although clinical studies have examined the relationship between SGLT2i therapy and myocardial deformation in adults with T2DM, the available evidence remains heterogeneous in terms of study design, sample size, patient population, baseline cardiovascular status, SGLT2i type, comparator group, treatment duration, echocardiographic protocol, and reported outcomes. Moreover, most evidence on SGLT2i has focused on clinical cardiovascular and renal outcomes, whereas fewer studies have specifically assessed subclinical myocardial function using LV GLS as a central imaging outcome [9,10]. Therefore, a focused synthesis of the available evidence is needed.

The primary objective of this systematic review was to synthesize the available evidence on the effects of SGLT2 inhibitors on LV GLS assessed by speckle-tracking echocardiography or equivalent strain imaging techniques in adults with T2DM. Secondary objectives were to describe the characteristics of the included studies, summarize related echocardiographic outcomes, and explore whether reported effects differed according to SGLT2 inhibitor type, cardiovascular phenotype, and study design.

## 2. Materials and Methods

### 2.1. Search Strategy

This study was designed as a systematic review aimed at synthesizing the available evidence regarding the effects of sodium-glucose cotransporter-2 inhibitors on left ventricular global longitudinal strain in adults with type 2 diabetes mellitus. This systematic review was conducted in accordance with the Preferred Reporting Items for Systematic Reviews and Meta-Analyses (PRISMA) guidelines [11] (File S4). The review protocol was registered in PROSPERO under registration number CRD420261383612.

A systematic literature search was conducted in PubMed, Scopus, Web of Science and Cochrane library to identify studies evaluating the effect of sodium-glucose cotransporter-2 (SGLT2) inhibitors on left ventricular global longitudinal strain (LV GLS) assessed by speckle-tracking echocardiography in adults with type 2 diabetes mellitus. These databases were chosen because they provide broad access to biomedical, clinical, and interdisciplinary scientific literature.

PubMed was used as a core biomedical database because of its extensive indexing of peer-reviewed medical and life sciences literature, including studies related to type 2 diabetes mellitus, cardiovascular disease, echocardiography, and pharmacological interventions. Web of Science was searched as a multidisciplinary citation database to ensure broader coverage of biomedical and cardiovascular research and to facilitate citation-based identification of relevant studies. Scopus was included because of its wide indexing of peer-reviewed scientific literature across medical, pharmacological, and clinical disciplines, thereby increasing the sensitivity of the search strategy. The Cochrane Library was searched as a specialized evidence-based medicine database to identify controlled trials, systematic reviews, and clinical study records relevant to SGLT2 inhibitor therapy and cardiovascular outcomes.

### 2.2. Review Question and PICO Framework

The search strategy was developed according to the PICO framework and was designed to identify studies evaluating the effect of SGLT2 inhibitors on left ventricular global longitudinal strain in adults with type 2 diabetes mellitus. A systematic electronic search was performed in PubMed, Scopus, Web of Science, and the Cochrane Library. The search combined controlled vocabulary, where available, and free-text terms related to the population, intervention, and outcome of interest. The search terms included synonyms and variations for type 2 diabetes mellitus, SGLT2 inhibitors, individual SGLT2 inhibitor agents, left ventricular global longitudinal strain, myocardial strain, and speckle-tracking echocardiography. The complete search strategies used for each database are provided in Supplementary File S1.

In PubMed, Medical Subject Headings (MeSH) and free-text terms were used. In Scopus, the search was conducted in the title, abstract, and keyword fields using the TITLE-ABS-KEY function. In Web of Science, topic-based terms were combined using Boolean operators. In the Cochrane Library, equivalent keyword combinations were used, including proximity operators where applicable.

The review question was formulated according to the PICO framework: in adults with type 2 diabetes mellitus, what is the effect of sodium-glucose cotransporter-2 inhibitors, compared with placebo, standard care, no SGLT2 inhibitor treatment, or other antihyperglycemic therapies, on left ventricular global longitudinal strain assessed by speckle-tracking echocardiography or equivalent strain imaging methods? The PICO components were defined as follows:

- Population: adults aged > 18 years with type 2 diabetes mellitus;
- Intervention: sodium-glucose cotransporter-2 inhibitors;
- Comparator: placebo, standard care, no SGLT2 inhibitor treatment, or other antihyperglycemic therapies;
- Outcome: left ventricular global longitudinal strain assessed by speckle-tracking echocardiography or equivalent strain imaging methods.

The search was limited to studies published in English from January 2015 onwards. In addition to database searching, the reference lists of included studies and relevant reviews were manually screened to identify additional eligible articles. Original clinical research articles were considered eligible, including randomized clinical trials, randomized controlled trials, placebo-controlled trials, prospective studies, prospective observational studies, prospective before–after studies, prospective cohort studies, and original clinical studies with a before–after design.

### 2.3. Study Selection

All records identified through the database searches were exported and imported into Rayyan web application (Rayyan Systems, Inc., Cambridge, MA, USA), which was used to organize the screening process and to identify duplicate records. Duplicates were removed before the eligibility assessment. Study selection was performed in two stages. First, titles and abstracts were independently screened by two reviewers according to the predefined inclusion and exclusion criteria. Second, the full texts of potentially eligible articles were retrieved and assessed in detail to confirm their eligibility for inclusion in the systematic review.

Disagreements or uncertain decisions were resolved through discussion and re-evaluation of the full-text articles. Studies were included only if they met the predefined eligibility criteria regarding population, intervention, comparator, outcomes, and study design.

Data from the included studies were extracted independently by two reviewers using a predefined data extraction form. Extracted variables included first author and year of publication, country, study design, study population, sample size, SGLT2 inhibitor type, comparator group, follow-up duration, LV GLS or LV strain assessment method, main echocardiographic outcomes, additional clinical or biochemical outcomes, and key findings. Extracted data were checked for consistency, completeness, and accuracy, and any disagreements or uncertainties were resolved through discussion and re-evaluation of the original full-text articles.

**Figure 1:**
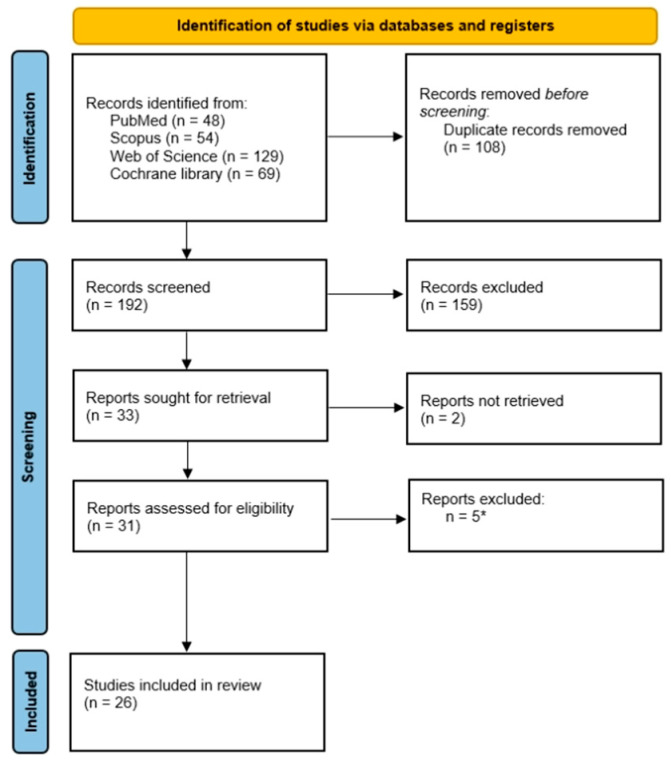
Study selection process according to PRISMA guidelines. * Reasons for exclusion are presented in Supplementary File S2.

The study selection process was documented using a PRISMA flow diagram, shown in Figure 1.

### 2.4. Eligibility Criteria

Studies were included if they enrolled adults with type 2 diabetes mellitus, evaluated treatment with an SGLT2 inhibitor, and reported left ventricular global longitudinal strain assessed by speckle-tracking echocardiography or an equivalent strain imaging method. Randomized controlled trials and original prospective or observational human studies were eligible.

Studies were considered eligible for inclusion if they met all of the following criteria:

- included adult participants with type 2 diabetes mellitus;
- evaluated treatment with a sodium-glucose cotransporter-2 inhibitor, including empagliflozin, dapagliflozin, canagliflozin, or ertugliflozin;
- reported left ventricular global longitudinal strain as an outcome;
- assessed LV GLS using speckle-tracking echocardiography or equivalent strain imaging methods;
- were original human studies published in full-text format;
- used a randomized controlled, prospective, or observational design;
- were published in the English language.

Studies enrolling populations with additional clinical conditions, such as heart failure, coronary artery disease, nonalcoholic fatty liver disease, or cancer, were considered eligible if they fulfilled the core PICO requirements and explicitly reported LV GLS or LV strain measured by speckle-tracking echocardiography or an equivalent strain imaging method in adults with type 2 diabetes mellitus.

Studies were excluded if they met any of the following criteria:

- review articles, systematic reviews, meta-analyses, editorials, letters, conference abstracts, guidelines, protocols, or case reports;
- animal studies, in vitro studies, or preclinical investigations;
- studies that did not evaluate a sodium-glucose cotransporter-2 inhibitor;
- studies that did not report left ventricular global longitudinal strain;
- studies that assessed only: left atrial strain, myocardial work, biomarkers, vascular or endothelial function, cardiac magnetic resonance imaging findings, without providing LV GLS data;
- studies in which the population of interest could not be distinguished from other diabetes types or mixed populations;
- non-English publications.

The eligibility criteria were designed to ensure that only studies directly relevant to the research question were included in the final synthesis.

### 2.5. Risk of Bias Assessment

The methodological quality of the included studies was assessed according to study design. Randomized controlled trials and randomized crossover studies were evaluated using the Cochrane Risk of Bias 2 (RoB 2) tool, while non-randomized studies were assessed using the Risk Of Bias In Non-randomized Studies of Interventions (ROBINS-I) tool.

The risk of bias assessment aimed to identify potential methodological limitations that could influence the interpretation of the findings. For randomized studies, the main domains considered included bias arising from the randomization process, deviations from intended interventions, missing outcome data, measurement of outcomes, and selection of reported results. For non-randomized studies, the main domains considered included bias due to confounding, selection of participants, classification of interventions, deviations from intended interventions, missing data, measurement of outcomes, and selection of reported results.

Particular attention was given to differences in baseline clinical characteristics, background therapy, comparator group, completeness of follow-up, and the objectivity and consistency of LV GLS assessment. The risk of bias assessment was used to support the interpretation of the evidence and to distinguish between stronger sources of evidence, such as randomized controlled trials, and studies with greater susceptibility to bias, particularly uncontrolled before–after and observational designs.

### 2.6. Data Analysis

A qualitative narrative synthesis was performed for all included studies. Data from the included studies were analyzed descriptively. Extracted variables included first author and year of publication, article title, journal, country, study design, study population, sample size, presence of type 2 diabetes mellitus and/or obesity in the study population, intervention (type of SGLT2 inhibitor), comparator, duration of follow-up, assessment of left ventricular global longitudinal strain or LV strain by speckle-tracking echocardiography or an equivalent strain imaging method, and main echocardiographic outcomes, with particular emphasis on left ventricular global longitudinal strain (LV GLS).

Because of the anticipated heterogeneity across studies in terms of design, patient characteristics, treatment duration, comparator groups, imaging methods, and outcome reporting, a meta-analysis was not planned as the primary approach. Therefore, no pooled effect estimates were calculated. Instead, the findings were synthesized narratively in order to provide a structured overview of the available evidence.

A quantitative meta-analysis was not performed because the included studies differed substantially in population phenotype, SGLT2 inhibitor type, comparator group, follow-up duration, imaging modality, strain software, reporting format, and availability of numerical GLS change data. Many studies reported only within-group changes, *p* values, or narrative conclusions rather than comparable baseline and follow-up mean values with standard deviations or between-group effect estimates. For this reason, a pooled estimate could have been statistically misleading and was not considered appropriate.

The direction of change in LV GLS, the presence or absence of statistically significant within-group or between-group differences, and the main echocardiographic and clinical outcomes reported by each study were summarized. Where appropriate, studies were grouped according to clinical setting and population characteristics, such as the presence of heart failure, preserved or reduced ejection fraction, coronary artery disease, or other relevant comorbidities, in order to facilitate comparison across studies.

Inter-vendor and inter-software variability were considered qualitatively during interpretation. Because LV GLS values may vary according to acquisition protocol, apical view selection, frame rate, tracking quality, segmental model, and vendor-specific algorithms, the review emphasized within-study changes and between-group comparisons rather than direct comparison of absolute GLS values across different studies.

No uniform threshold for a clinically meaningful improvement in LV GLS was applied across studies because the original articles used different definitions, strain platforms, segmental models, and statistical reporting approaches. When available, the synthesis considered absolute GLS values, direction of change, statistical significance, and between-group differences; however, conclusions were interpreted cautiously because small numerical changes may have different clinical meaning depending on baseline GLS, LVEF, clinical phenotype, and vendor-specific measurement variability.

Formal assessment of publication bias was not performed because no meta-analysis was conducted and the included studies were clinically and methodologically heterogeneous. A formal GRADE assessment was not performed; therefore, certainty of evidence was interpreted narratively based on study design, risk of bias, consistency of findings, and clinical heterogeneity.

Use of generative artificial intelligence ChatGPT version 5.1 (OpenAI, San Francisco, CA, USA) was used only for English-language editing, grammar correction, and readability refinement after drafting and translation of the manuscript. The tool was not used to select studies, extract data, assess risk of bias, perform analyses, synthesize evidence, interpret results, or formulate conclusions. All scientific and methodological decisions were made and verified by the authors.

## 3. Results

### 3.1. Overview of Selected Studies

The literature search identified studies evaluating the effect of SGLT2 inhibitors on LV GLS or LV strain assessed by speckle-tracking echocardiography or equivalent strain imaging methods in adults with type 2 diabetes mellitus.

The database search identified 300 records across PubMed, Scopus, Web of Science, and the Cochrane Library. After deduplication, 108 duplicate records were removed, leaving 192 records for title and abstract screening. Of these, 159 records were excluded, and 33 reports were sought for full-text retrieval. Two reports could not be retrieved, while 31 full-text articles were assessed for eligibility. Five articles were excluded, and 26 studies were included in the final synthesis (Figure 1).

The 26 included studies were original human investigations evaluating at least one SGLT2 inhibitor and reporting LV GLS or LV strain in adults with T2DM [12,13,14,15,16,17,18,19,20,21,22,23,24,25,26,27,28,29,30,31,32,33,34,35,36,37]. Most studies assessed LV GLS by speckle-tracking echocardiography, while one study used cardiac magnetic resonance feature-tracking to evaluate LV strain [19]. The excluded full-text articles did not fully meet the predefined eligibility criteria, mainly because the independent effect of SGLT2 inhibitor therapy could not be isolated or because SGLT2 inhibitors were evaluated in direct comparison with, or in combination with, other glucose-lowering therapies such as GLP-1 receptor agonists, DPP-4 inhibitors, or insulin [38,39,40,41,42]. Detailed reasons for exclusion are presented in Supplementary File S2.

The included studies represented at least 2380 participants and comprised randomized controlled trials, randomized crossover studies, prospective observational studies, retrospective observational studies, before–after studies, controlled observational studies, and post hoc or secondary analyses [12,13,14,15,16,17,18,19,20,21,22,23,24,25,26,27,28,29,30,31,32,33,34,35,36,37]. Sample sizes varied considerably, ranging from small single-center studies with 25–31 participants to larger cohorts, including the propensity score-matched analysis by Wang et al. and the prospective cohort study by Kümet et al. [13,20,22,27,36].

The studies were published between 2020 and 2026 and were conducted across several countries, including Spain, China, the Republic of Korea, Australia, Japan, Canada, Italy, Turkey, Taiwan, Denmark, Iran, Egypt, and Croatia [12,13,14,15,16,17,18,19,20,21,22,23,24,25,26,27,28,29,30,31,32,33,34,35,36,37]. The included studies showed substantial clinical and methodological heterogeneity in terms of patient population, baseline cardiovascular status, study design, comparator group, SGLT2 inhibitor type, echocardiographic protocol, and follow-up duration. A detailed overview of the main characteristics of the included studies, including study design, population, sample size, intervention, comparator, main echocardiographic outcomes, key findings, and follow-up duration, is provided in Table 1.

Regarding study design, several studies used prospective observational, prospective follow-up, or before–after designs without a separate control group [12,13,16,17,20,21,22,24,26,27,30,32,34,37]. Randomized or randomized crossover designs were used in the Ertu-GLS trial, the LEAVE-DM trial, the EMPA-HEART CardioLink-6 strain substudy, the randomized crossover trial by Eickhoff et al., the randomized single-blind trial comparing empagliflozin with pioglitazone, and the ELUCIDATE randomized controlled study [14,15,19,25,29,33]. Other studies used controlled observational comparisons, active-comparator designs, propensity score matching, or secondary/post hoc analyses [18,23,28,31,35,36].

The included populations also differed considerably. Some studies enrolled adults with T2DM without known cardiovascular disease, without established cardiac disease, or with preserved left ventricular ejection fraction [13,22,24,26,28,30,32,36,37]. Other studies focused on patients with pre-heart failure, stage B heart failure, asymptomatic heart failure, chronic heart failure, heart failure with reduced ejection fraction, heart failure with mildly reduced ejection fraction, coronary artery disease, acute coronary syndrome, albuminuria, non-alcoholic fatty liver disease, hypertension, or doxorubicin-related cardiotoxicity risk [14,15,16,17,18,19,20,21,23,25,27,29,31,33,34,35,37]. The evaluated SGLT2 inhibitors included dapagliflozin, empagliflozin, ertugliflozin, canagliflozin, and mixed SGLT2 inhibitor regimens [12,13,14,15,16,17,18,19,20,21,22,23,24,25,26,27,28,29,30,31,32,33,34,35,36,37].

Follow-up duration ranged from 12 weeks to 24 months. Three-month or 12-week follow-up was reported in studies evaluating empagliflozin, dapagliflozin, or mixed SGLT2 inhibitor therapy [21,23,25,26]. Most studies assessed outcomes after approximately 6 months or 24 weeks [12,13,14,15,17,18,19,20,22,24,27,28,29,30,31,32,33,34,35,36,37], while one study followed patients for 12 months [16]. The LEAVE-DM trial included follow-up assessments at both 6 and 24 months [15].

Because follow-up duration, patient phenotype, and comparator design differed across studies, no clear dose-duration or time-response relationship could be established. Improvements were reported in some short-term studies of approximately 12 weeks or 3 months, as well as in several 6-month studies, whereas longer follow-up did not consistently translate into larger GLS changes. Therefore, treatment duration should be interpreted together with baseline myocardial dysfunction, background therapy, and study design.

### 3.2. Study Populations

All included studies enrolled adults with type 2 diabetes mellitus and assessed clinically diverse populations across different cardiovascular and metabolic contexts [12,13,14,15,16,17,18,19,20,21,22,23,24,25,26,27,28,29,30,31,32,33,34,35,36,37]. Several studies focused on adults with T2DM without overt cardiovascular disease, without established cardiac disease, or with preserved left ventricular ejection fraction, aiming to evaluate subclinical myocardial dysfunction before the development of clinically apparent heart failure [13,22,24,26,28,30,32,36,37]. Other studies included patients with higher cardiovascular risk or established cardiovascular conditions, such as acute coronary syndrome, stable coronary artery disease, pre-heart failure, stage B heart failure, asymptomatic heart failure, chronic heart failure, heart failure with reduced ejection fraction, heart failure with mildly reduced ejection fraction, or diabetes-related cardiomyopathy [14,15,16,17,18,19,20,21,31,33,34,35].

Several studies examined more specific clinical subgroups. One study enrolled adults with T2DM and albuminuria receiving renin–angiotensin–aldosterone system blockade [25], while another included adults with T2DM and non-alcoholic fatty liver disease without established atherosclerotic cardiovascular disease [29]. One study evaluated newly diagnosed breast cancer patients with T2DM receiving doxorubicin-based chemotherapy, addressing the potential cardioprotective effect of dapagliflozin in the context of chemotherapy-related cardiotoxicity risk [23]. Additional studies stratified or selected patients according to body mass index, diabetes duration, hypertension, coronary artery disease status, or the presence of subclinical echocardiographic abnormalities [22,34,36,37]. Overall, the included studies demonstrated marked heterogeneity in baseline cardiovascular status, comorbidities, and clinical setting, ranging from asymptomatic patients with preserved ejection fraction to patients with established heart failure or complex cardiometabolic risk profiles [12,13,14,15,16,17,18,19,20,21,22,23,24,25,26,27,28,29,30,31,32,33,34,35,36,37].

### 3.3. Interventions and Comparators

The included studies evaluated different SGLT2 inhibitors, with dapagliflozin and empagliflozin being the most frequently investigated agents. Dapagliflozin was assessed as monotherapy or add-on therapy in several clinical contexts: in adults with T2DM and chronic heart failure [17], in patients with preserved ejection fraction and cardiac structural abnormalities [27], in T2DM patients with albuminuria in a randomized crossover trial [25], in asymptomatic T2DM patients in the ELUCIDATE randomized controlled study [33], and in patients with stage B heart failure in the LEAVE-DM trial [15]. Dapagliflozin was also evaluated in more specific populations, including T2DM patients receiving doxorubicin-based chemotherapy for breast cancer [23], patients with T2DM undergoing assessment of epicardial adipose tissue and LV GLS [13], and translational human and experimental models of diabetes-related myocardial dysfunction [24].

Empagliflozin was evaluated in several studies with distinct clinical designs. Oka et al. assessed empagliflozin in patients with diabetes mellitus-related cardiomyopathy [16], while Lan et al. evaluated empagliflozin initiated after acute coronary syndrome in patients with T2DM [18]. Moses et al. assessed the effect of empagliflozin on LV strain in a substudy of the EMPA-HEART CardioLink-6 randomized trial in patients with T2DM and stable coronary artery disease [19]. Nesti et al. compared empagliflozin with sitagliptin in adults with T2DM without clinical or echocardiographic evidence of heart disease [28], and Attaran et al. compared empagliflozin with pioglitazone in patients with T2DM and non-alcoholic fatty liver disease without established cardiovascular disease [29]. Other empagliflozin studies used before–after designs in patients with diabetes without cardiovascular disease [26], patients with normal ejection fraction and cardiovascular risk factors [32], and patients with T2DM and hypertension [37].

Other SGLT2 inhibitors were less frequently studied. Ertugliflozin was evaluated in the Ertu-GLS randomized placebo-controlled trial, which included adults with T2DM and pre-heart failure [14]. Canagliflozin was assessed by Kuo et al. in patients with T2DM receiving canagliflozin 100 mg/day, using a before–after design to evaluate cardiac remodeling and hemodynamic parameters [30]. Several studies evaluated mixed SGLT2 inhibitor therapy rather than a single agent. Gamaza-Chulián et al. included patients receiving empagliflozin, dapagliflozin, canagliflozin, or ertugliflozin [12]. Palmiero/Cesaro et al. assessed SGLT2 inhibitor therapy, mainly empagliflozin, in patients with T2DM and HFrEF [20]. Savcılıoglu et al. evaluated empagliflozin or dapagliflozin added to optimized HFrEF therapy [21], while Kümet et al. included patients newly initiated on empagliflozin or dapagliflozin according to clinical indication [22]. Mixed or clinically selected SGLT2 inhibitor therapy was also assessed in studies by El-Saied et al., Biter et al., Grubić Rotkvić et al., and Wang et al. [31,34,35,36].

Comparator groups differed substantially between studies. Placebo was used in randomized controlled designs, including the Ertu-GLS trial of ertugliflozin [14], the LEAVE-DM trial of dapagliflozin [15], the EMPA-HEART CardioLink-6 substudy of empagliflozin [19], and the randomized crossover trial of dapagliflozin by Eickhoff et al. [25]. Active comparators were used in several studies: Nesti et al. compared empagliflozin with sitagliptin [28], Attaran et al. compared empagliflozin with pioglitazone [29], and Ozturk et al. compared dapagliflozin added to metformin with other non-dapagliflozin oral antidiabetic therapy during doxorubicin treatment [23]. Other controlled studies compared SGLT2 inhibitor therapy with non-SGLT2 inhibitor treatment, standard care, or treatment without SGLT2 inhibitors [12,16,18,31,35,36].

A considerable number of studies did not include a separate comparator group and instead used a before–after design, comparing echocardiographic parameters before and after SGLT2 inhibitor initiation. This approach was used in studies evaluating dapagliflozin [13,17,24,27], empagliflozin [26,32,37], canagliflozin [30], and mixed SGLT2 inhibitor therapy [20,21,22,34]. These uncontrolled designs were mainly assessed within-patient changes in LV GLS, LVEF, LV mass, diastolic function, left atrial function, myocardial work indices, epicardial adipose tissue, NT-proBNP, or other cardiometabolic markers after treatment initiation. Overall, the intervention and comparator frameworks were highly heterogeneous, ranging from placebo-controlled randomized trials to active-comparator studies, controlled observational studies, and uncontrolled before–after analyses, which should be considered when interpreting the strength and consistency of the reported effects [12,13,14,15,16,17,18,19,20,21,22,23,24,25,26,27,28,29,30,31,32,33,34,35,36,37].

### 3.4. Assessment of LV GLS

All included studies assessed LV GLS or LV strain as the main marker of subclinical left ventricular systolic function [12,13,14,15,16,17,18,19,20,21,22,23,24,25,26,27,28,29,30,31,32,33,34,35,36,37]. Most studies used two-dimensional speckle-tracking echocardiography, while Moses et al. used cardiac magnetic resonance feature-tracking in the EMPA-HEART CardioLink-6 substudy [19]. In the echocardiographic studies, LV GLS was generally derived from standard apical views and calculated as the average peak systolic longitudinal strain across LV myocardial segments.

Several studies reported additional parameters of LV structure and systolic function. LVEF was commonly assessed together with LV GLS, while LV mass, LV mass index, or remodeling parameters were reported in studies such as Gamaza-Chulián et al., Lim et al., Marwick et al., Moses et al., Cortés et al., Kuo et al., Lin et al., and Wang et al. [12,14,15,19,27,30,33,36].

Diastolic function was also frequently evaluated. Tanaka et al., Lan et al., Eickhoff et al., Cortés et al., Attaran et al., Kuo et al., El-Saied et al., Wang et al., and Karaduman et al. reported parameters such as E/e′, E/A ratio, tissue Doppler e′ velocity, or related indices of LV filling pressure [17,18,25,27,29,30,31,36,37]. Left atrial structure or function was assessed in selected studies, including Marwick et al., Lan et al., Palmiero/Cesaro et al., Savcılıoglu et al., Ozturk et al., Cortés et al., El-Saied et al., Cheng et al., and Karaduman et al. [15,18,20,21,23,27,31,32,37].

Advanced imaging and mechanistic outcomes were less consistently reported. Song et al. and Wang et al. assessed epicardial adipose tissue thickness [13,36]. Palmiero/Cesaro et al., Dural et al., and Cheng et al. evaluated myocardial work or myocardial energetic efficiency [20,26,32]. Savcılıoglu et al. and Palmiero/Cesaro et al. also reported right ventricular or pulmonary pressure-related parameters [20,21]. Biomarkers or mechanistic markers, including NT-proBNP, inflammatory or oxidative stress markers, asprosin, MMP-1, TIMP-1, or experimental markers of endoplasmic reticulum stress, fibrosis, and apoptosis, were reported in selected studies [21,23,24,25,31,34,35,37].

Overall, LV GLS was the common imaging outcome across all included studies, but the associated echocardiographic, biomarker, and mechanistic assessments varied substantially according to study design, clinical setting, and research focus [12,13,14,15,16,17,18,19,20,21,22,23,24,25,26,27,28,29,30,31,32,33,34,35,36,37].

### 3.5. Effects of SGLT2 Inhibitors on LV GLS

Overall, most included studies reported favorable changes in LV GLS or LV strain after SGLT2 inhibitor therapy, although the consistency of this effect differed according to study design, comparator group, baseline cardiovascular status, and clinical phenotype [12,13,14,15,16,17,18,19,20,21,22,23,24,25,26,27,28,29,30,31,32,33,34,35,36,37].

In observational or before–after studies, Gamaza-Chulián et al. reported improved longitudinal strain and reduced indexed LV mass after SGLT2 inhibitor therapy [12]. Song et al. found that dapagliflozin improved LV GLS and reduced epicardial adipose tissue thickness [13]. In the Ertu-GLS randomized trial, Lim et al. showed that ertugliflozin significantly improved LV GLS, LV mass index, and LVEF compared with placebo [14]. In contrast, Marwick et al. reported improvements in diastolic function, left atrial function, and exercise capacity with dapagliflozin, but no significant between-group improvement in GLS [15]. Oka et al. found that empagliflozin improved LV GLS in diabetes-related cardiomyopathy, particularly in the early disease phase [16], while Tanaka et al. reported improved LV longitudinal function after dapagliflozin in patients with T2DM and chronic heart failure, especially in HFpEF [17].

Lan et al. found that empagliflozin after acute coronary syndrome reduced LV mass and improved diastolic parameters, without a significant between-group change in LV GLS [18]. Moses et al., in the EMPA-HEART CardioLink-6 strain substudy, reported no significant improvement in CMR-derived LV peak GLS with empagliflozin compared with placebo [19]. Palmiero/Cesaro et al. reported improvements in GLS, LVEF, myocardial work, left atrial function, right ventricular function, and pulmonary pressure-related parameters in patients with T2DM and HFrEF [20]. Savcılıoglu et al. found improved LV GLS and left atrial strain after empagliflozin or dapagliflozin in patients with HFrEF and T2DM, despite unchanged LVEF [21]. Kümet et al. showed that SGLT2 inhibitors improved GLS similarly in normal-weight and overweight or obese patients with T2DM [22].

In a cardio-oncology population, Ozturk et al. reported improved LV GLS and reduced NT-proBNP with dapagliflozin in breast cancer patients with T2DM receiving doxorubicin [23]. Shih et al. found that dapagliflozin improved longitudinal strain and diastolic function, with experimental data suggesting reduced endoplasmic reticulum stress, fibrosis, and apoptosis [24]. Eickhoff et al. reported no significant effect of dapagliflozin on GLS in a randomized crossover trial, although exploratory diastolic function parameters improved [25]. Dural et al. found improved LV GLS and myocardial mechano-energetic efficiency after empagliflozin [26], while Cortés et al. reported improved LV GLS and cardiac structural parameters after dapagliflozin in patients with preserved ejection fraction [27].

Active-comparator studies showed mixed findings. Nesti et al. found neutral overall effects of empagliflozin compared with sitagliptin, although GLS improved in patients with baseline subclinical dysfunction [28]. Attaran et al. reported GLS improvement in both empagliflozin and pioglitazone groups, without a significant between-group difference [29]. Kuo et al. found favorable changes in LV GLS, cardiac remodeling, and hemodynamic or diastolic parameters after canagliflozin [30]. El-Saied et al. reported improved LV GLS, left atrial function, and diastolic parameters in patients with T2DM and HFmrEF treated with SGLT2 inhibitors [31]. Cheng et al. found improvements in LV GLS, myocardial work indices, right ventricular strain, and left atrial strain after empagliflozin [32].

In the ELUCIDATE randomized controlled study, Lin et al. reported that dapagliflozin add-on therapy improved cardiac remodeling and mechanical function, including GLS [33]. Biter et al. found improvement in LV longitudinal strain during follow-up regardless of coronary artery disease status [34]. Grubić Rotkvić et al. reported favorable effects of SGLT2 inhibitor therapy on myocardial function and cardiac load in patients with T2DM and asymptomatic heart failure [35]. Wang et al., using propensity score matching, found better GLS and a lower rate of subclinical LV systolic dysfunction with SGLT2 inhibitor therapy regardless of diabetes duration [36]. Karaduman et al. reported improved GLS after empagliflozin in patients with T2DM and hypertension, together with improved diastolic parameters and reductions in NT-proBNP, blood pressure, waist circumference, and BMI [37].

Taken together, the evidence suggests that SGLT2 inhibitors may improve LV GLS in many adults with T2DM, particularly in observational and before–after studies. However, randomized placebo-controlled and active-comparator studies showed less consistent results, indicating that baseline myocardial dysfunction, comparator choice, follow-up duration, imaging method, and clinical phenotype may influence the observed effect on myocardial deformation [12,13,14,15,16,17,18,19,20,21,22,23,24,25,26,27,28,29,30,31,32,33,34,35,36,37].

Across agents, the available evidence did not allow a reliable ranking of dapagliflozin, empagliflozin, ertugliflozin, canagliflozin, or mixed SGLT2i regimens. Apparent differences between agents were strongly confounded by differences in study design, comparator group, patient population, sample size, baseline cardiovascular status, treatment duration, and imaging method. Thus, the review supports a possible class-associated effect on myocardial deformation rather than the superiority of a specific SGLT2 inhibitor.

### 3.6. Additional Echocardiographic and Clinical Outcomes

Beyond LV GLS, several studies reported changes in cardiac structure and remodeling. Gamaza-Chulián et al. observed a reduction in indexed LV mass after SGLT2 inhibitor therapy [12]. Lim et al. found that ertugliflozin improved LV mass index, LVEF, and E/e′ in patients with T2DM and pre-heart failure [14]. Marwick et al. reported improved diastolic function, left atrial function, and exercise capacity after dapagliflozin, despite no significant between-group GLS improvement [15]. Lan et al. observed reduced LV mass index and improved diastolic parameters after empagliflozin following acute coronary syndrome [18]. Moses et al. discussed LV mass regression in the parent EMPA-HEART CardioLink-6 trial, although the strain substudy did not show a significant GLS benefit [19]. Cortés et al., Kuo et al., Lin et al., and Wang et al. also reported favorable structural, remodeling, or hemodynamic changes after SGLT2 inhibitor therapy [27,30,33,36].

Changes in LVEF were inconsistent. Palmiero/Cesaro et al. reported improved LVEF in patients with T2DM and HFrEF [20], while several other studies showed improved LV GLS or strain-based parameters despite unchanged LVEF [13,17,21,24,25,26,32,37]. This supports the role of LV GLS as a more sensitive marker of subclinical myocardial improvement than conventional systolic function.

Diastolic function was frequently assessed and often improved. Improvements in E/e′, E/A ratio, tissue Doppler e′ velocity, or related filling pressure parameters were reported in studies of patients with chronic heart failure, acute coronary syndrome, stage B heart failure, albuminuria, preserved ejection fraction, NAFLD, HFmrEF, asymptomatic heart failure, and hypertension [15,17,18,25,27,29,30,31,35,36,37]. Left atrial outcomes were assessed in fewer studies, with improvements in left atrial volume, ejection fraction, or strain reported after dapagliflozin, empagliflozin, or mixed SGLT2 inhibitor therapy [15,18,20,21,23,31,32,37].

Advanced imaging and mechanistic outcomes were reported less consistently. Palmiero/Cesaro et al., Dural et al., and Cheng et al. found improvements in myocardial work indices or myocardial mechano-energetic efficiency [20,26,32]. Oka et al. used CMR-derived extracellular volume to distinguish early from advanced diabetes-related cardiomyopathy and found greater empagliflozin-related GLS improvement in early disease [16]. Shih et al. combined clinical and experimental data and reported reduced endoplasmic reticulum stress, fibrosis, and apoptosis after dapagliflozin [24]. Song et al. showed that dapagliflozin reduced epicardial adipose tissue thickness and that this reduction was independently associated with LV GLS improvement [13], while Wang et al. also included epicardial adipose tissue among the assessed echocardiographic parameters [36].

Clinical, functional, and biochemical outcomes varied across studies. Reductions in NT-proBNP were reported in studies involving HFrEF, cardio-oncology, HFmrEF, and hypertension populations [21,23,31,37], whereas Biter et al. found improved strain without a significant NT-proBNP change [34]. Nesti et al. reported neutral overall effects of empagliflozin on LV GLS and VO_2_peak, although GLS improved in patients with baseline subclinical dysfunction [28]. Several studies also described improvements in cardiometabolic markers, including HbA1c, body weight, BMI, waist circumference, blood pressure, uric acid, proteinuria, lipid parameters, and inflammatory or oxidative stress markers [13,14,21,22,24,30,31,32,35,36,37].

Overall, these findings suggest that the effects of SGLT2 inhibitors may extend beyond LV GLS and involve LV remodeling, diastolic function, left atrial function, myocardial work, adiposity-related parameters, biomarkers, functional capacity, and cardiometabolic risk markers. However, these outcomes were assessed inconsistently across studies, limiting direct comparison and supporting a cautious interpretation of the broader echocardiographic and clinical effects [12,13,14,15,16,17,18,19,20,21,22,23,24,25,26,27,28,29,30,31,32,33,34,35,36,37].

### 3.7. Risk of Bias

Risk of bias was assessed according to study design. Randomized studies were evaluated using the Cochrane RoB 2 tool, while non-randomized studies were assessed using ROBINS-I. Overall, randomized and placebo-controlled studies had a lower risk of bias than observational and before–after studies. Detailed study-level risk of bias assessments and supporting justifications are provided in Supplementary File S3.

The main sources of potential bias in non-randomized studies were confounding, participant selection, absence of a control group, and differences in background therapy or clinical characteristics. Single-arm before–after studies were particularly limited because changes in LV GLS could not be clearly attributed to SGLT2 inhibitor therapy alone.

Overall, although many studies reported favorable changes in LV GLS, the evidence should be interpreted cautiously due to methodological heterogeneity and the predominance of observational or uncontrolled study designs.

## 4. Discussion

### 4.1. Summary of the Main Findings

This systematic review synthesized the available evidence on the effects of sodium-glucose cotransporter-2 inhibitors (SGLT2i) on left ventricular global longitudinal strain (LV GLS) or LV strain in adults with type 2 diabetes mellitus (T2DM). Across the 26 included studies, the overall direction of evidence suggested that SGLT2i therapy may be associated with favorable changes in myocardial deformation, particularly in observational, before–after, and prospective follow-up studies [12,13,16,17,20,21,22,23,24,26,27,30,31,32,34,35,36,37]. Improvements in LV GLS were reported with different agents, including dapagliflozin [13,17,23,24,27,33], empagliflozin [16,26,32,37], canagliflozin [30], ertugliflozin [14], and mixed SGLT2i regimens [12,20,21,22,31,34,35,36].

Favorable changes were observed across a broad range of clinical phenotypes. Several studies reported improvement in LV GLS among adults with T2DM and preserved ejection fraction, no overt cardiovascular disease, or subclinical cardiac dysfunction [13,22,24,26,27,30,32,36,37]. Other studies suggested potential benefit in higher-risk populations, including diabetes-related cardiomyopathy [16], chronic heart failure or HFrEF [17,20,21], HFmrEF [31], asymptomatic heart failure [35], acute coronary syndrome or coronary artery disease [18,19,34], and patients at risk of doxorubicin-related cardiotoxicity [23]. These findings suggest that the potential effect of SGLT2i on myocardial deformation may extend across different stages of diabetes-related cardiovascular involvement.

However, the randomized and controlled evidence was less consistent. The Ertu-GLS randomized placebo-controlled trial showed significant improvement in LV GLS with ertugliflozin compared with placebo in adults with T2DM and pre-heart failure [14], and the ELUCIDATE randomized controlled study reported improved cardiac remodeling and mechanical function after dapagliflozin add-on therapy [33]. In contrast, the LEAVE-DM trial did not demonstrate a significant between-group improvement in GLS, despite favorable effects on diastolic function, left atrial function, and exercise capacity [15]. Similarly, the EMPA-HEART CardioLink-6 strain substudy found no significant improvement in CMR-derived LV peak GLS with empagliflozin compared with placebo [19], while the randomized crossover trial by Eickhoff et al. showed no significant effect of dapagliflozin on GLS despite minor improvement in diastolic function [25].

Active-comparator studies also yielded mixed results. In the EMPA-HEART trial, empagliflozin had neutral overall effects on LV GLS and peak oxygen uptake compared with sitagliptin, although GLS improved in the subgroup with baseline subclinical dysfunction [28]. Attaran et al. reported that GLS improved in both the empagliflozin and pioglitazone groups, without a significant between-group difference [29]. These findings indicate that improvements in LV GLS may not be specific to SGLT2i therapy in all clinical settings and may depend on baseline myocardial dysfunction, comparator treatment, metabolic changes, and study design.

Therefore, the main conclusion of this review is that SGLT2i therapy may be associated with improvement in LV GLS in adults with T2DM, especially in observational and before–after studies. Nevertheless, the evidence does not definitively establish that SGLT2i improve LV GLS in all adults with T2DM. The findings should be interpreted cautiously because the included studies differed substantially in study design, comparator group, SGLT2i agent, follow-up duration, imaging modality, echocardiographic protocol, and baseline cardiovascular phenotype [12,13,14,15,16,17,18,19,20,21,22,23,24,25,26,27,28,29,30,31,32,33,34,35,36,37]. Larger randomized studies using standardized strain imaging protocols are needed to clarify the magnitude, consistency, and clinical significance of SGLT2i-related changes in LV GLS.

### 4.2. Clinical Relevance of LV GLS Improvement

LV GLS is clinically relevant because it can detect subtle myocardial dysfunction before changes in LVEF become apparent and provides additional diagnostic and prognostic information beyond conventional systolic assessment [9]. This is especially important in T2DM, where myocardial impairment may occur despite preserved LVEF and in the absence of overt heart failure symptoms [10].

In several included studies, treatment with SGLT2 inhibitors was associated with improved LV GLS despite unchanged LVEF, supporting the sensitivity of strain imaging for detecting early functional changes [13,17,21,24,25,26,32,37]. These changes were often accompanied by improvements in related parameters, such as epicardial adipose tissue, myocardial work indices, diastolic function, left atrial function, NT-proBNP, blood pressure, body weight, or glycemic control [13,20,23,31,32,37]. Therefore, improvement in LV GLS may reflect broader favorable effects on myocardial performance and cardiometabolic status.

However, not all controlled studies showed a significant between-group improvement in GLS [15,19,25,28,29]. Thus, although LV GLS appears useful for detecting early myocardial changes, further randomized studies are needed to determine whether SGLT2i-related strain improvement translates into reduced heart failure progression or cardiovascular events in adults with T2DM.

Importantly, the included GLS-focused studies were not designed or powered to determine whether changes in LV GLS translate into fewer heart failure hospitalizations, cardiovascular events, or deaths. Therefore, LV GLS should be interpreted as a surrogate imaging outcome that may indicate early myocardial change, while its relationship with hard clinical outcomes in SGLT2i-treated adults with T2DM remains to be proven.

### 4.3. Biological Plausibility and Potential Mechanisms

The potential improvement in LV GLS after SGLT2i therapy is biologically plausible, as these agents have demonstrated cardiovascular benefits that extend beyond glucose lowering, particularly in relation to heart failure outcomes [7,8]. Several mechanisms may contribute to improved myocardial deformation, including natriuresis, osmotic diuresis, reduced preload and afterload, improved myocardial energetics, enhanced mitochondrial function, reduced inflammation and oxidative stress, antifibrotic effects, and improved cardiorenal interactions [43].

These mechanisms may explain why strain-based parameters improved in several studies even when LVEF remained unchanged [13,17,21,24,25,26,32,37]. In the included studies, LV GLS improvement was often accompanied by favorable changes in epicardial adipose tissue, blood pressure, body weight, HbA1c, NT-proBNP, diastolic function, left atrial function, or myocardial work indices [13,20,23,31,32,37]. This suggests that LV GLS may capture early myocardial effects of SGLT2i that are not yet reflected by conventional systolic measures.

Biomarkers of myocardial remodeling, fibrosis, inflammation, oxidative stress, or extracellular matrix turnover were not reported consistently across studies. Some studies included NT-proBNP, asprosin, MMP-1, TIMP-1, oxidative stress markers, or translational markers of endoplasmic reticulum stress, fibrosis, and apoptosis, but these data were heterogeneous and insufficient for a unified mechanistic synthesis.

The observed GLS changes cannot be assumed to reflect a direct myocardial effect alone. In several studies, SGLT2i therapy was accompanied by reductions in HbA1c, body weight, blood pressure, NT-proBNP, epicardial adipose tissue, or inflammatory and oxidative stress markers, each of which may influence loading conditions, myocardial energetics, ventricular-arterial interaction, and strain-derived measurements. Consequently, improved LV GLS may represent the integrated effect of hemodynamic unloading, metabolic improvement, vascular effects, and direct myocardial signaling rather than a single pathway.

### 4.4. Heterogeneity and Interpretation of the Evidence

A major feature of the included evidence was the substantial clinical and methodological heterogeneity. The studies enrolled patients with different cardiovascular and metabolic profiles, including adults with T2DM without overt cardiovascular disease or with preserved LVEF [13,22,24,26,27,30,32,36,37], pre-heart failure or stage B heart failure [14,15], diabetes-related cardiomyopathy [16], chronic heart failure or HFrEF [17,20,21], HFmrEF [31], coronary artery disease or acute coronary syndrome [18,19,34], NAFLD [29], asymptomatic heart failure [35], and doxorubicin-related cardiotoxicity risk [23]. These populations differ in baseline myocardial function, cardiovascular risk, comorbidities, and potential for reverse remodeling.

This heterogeneity may partly explain the variability in LV GLS response. Patients with impaired baseline myocardial deformation may have greater potential for improvement, whereas those with preserved or near-normal strain may show smaller changes during short follow-up. Differences in comparator groups also limited direct comparison, as studies used placebo [14,15,19,25], active comparators such as sitagliptin or pioglitazone [28,29], non-SGLT2i treatment or standard care [12,16,18,23,31,35,36], or uncontrolled before–after designs [13,17,20,21,22,24,26,27,30,32,34,37].

The discrepancy between observational and randomized evidence is important. Most observational and before–after studies suggested improvement in LV GLS [12,13,16,17,20,21,22,23,24,26,27,30,31,32,34,35,36,37], but these designs cannot fully separate the effect of SGLT2i from changes in glycemic control, blood pressure, body weight, background therapy, or regression to the mean. Randomized and controlled studies provided stronger evidence but showed mixed results, with positive findings in some trials [14,33] and neutral or less consistent findings in others [15,19,25,28,29]. Therefore, the current evidence supports a possible beneficial association between SGLT2i therapy and LV GLS improvement, but not a definitive effect across all T2DM populations.

This discrepancy may reflect several factors. Observational and before-after studies are more likely to include patients with active cardiometabolic optimization, larger concurrent changes in glycemic control, weight, blood pressure, or background therapy, and regression to the mean. Randomized trials, by contrast, more directly compare SGLT2i therapy with placebo or active treatment and may include patients with less impaired baseline GLS or shorter follow-up for detecting structural myocardial change. These differences likely contributed to the weaker and more variable treatment signal in randomized and controlled studies.

### 4.5. Additional Echocardiographic and Biochemical Findings

Several included studies reported favorable changes in cardiac parameters beyond LV GLS, including LV mass index, LV remodeling indices, diastolic function, E/e′ ratio, left atrial structure and function, myocardial work indices, NT-proBNP, and epicardial adipose tissue thickness [12,13,14,15,18,20,21,23,26,27,31,32,36,37]. These outcomes are relevant to the interpretation of myocardial structure and function because conventional echocardiographic parameters, diastolic indices, myocardial work, and epicardial adipose tissue are established tools or markers used in cardiac assessment [44,45,46,47].

Changes in LVEF were less consistent than changes in LV GLS. In several included studies, LV GLS improved while LVEF remained unchanged, supporting the greater sensitivity of strain imaging for detecting early myocardial functional changes [13,17,21,24,25,26,32,37]. Diastolic function parameters, including E/e′, tissue Doppler e′ velocity, E/A ratio, and left atrial indices, were also frequently assessed across the included studies [15,17,18,25,27,29,30,31,36,37].

Advanced parameters provided additional mechanistic insight within the selected included studies. Myocardial work indices were assessed in studies by Palmiero/Cesaro et al., Dural et al., and Cheng et al. [20,26,32]. Epicardial adipose tissue thickness was evaluated by Song et al. and Wang et al. [13,36]. Biochemical and clinical outcomes also varied, with reductions in NT-proBNP reported in selected populations [21,23,31,37], while Biter et al. found unchanged NT-proBNP despite improved strain parameters [34]. Improvements in body weight, BMI, blood pressure, HbA1c, inflammatory or oxidative stress markers, and other cardiometabolic variables were reported in several studies [13,21,22,24,30,32,35,36,37].

Overall, these additional findings suggest a potential multidimensional cardiovascular effect of SGLT2i, involving myocardial structure, filling pressures, atrial function, myocardial energetics, adiposity-related remodeling, biomarkers, and cardiometabolic risk markers. However, these outcomes were not assessed uniformly across the included studies, limiting direct comparison.

### 4.6. Risk of Bias and Certainty of Evidence

The certainty of the evidence is limited by the methodological quality of the included studies. Risk of bias was assessed using RoB 2 for randomized studies and ROBINS-I for non-randomized studies [48,49]. Randomized and placebo-controlled studies generally provided stronger evidence, but their findings were not fully consistent: some trials reported improvement in LV GLS, whereas others found neutral effects despite favorable changes in other cardiac parameters [14,15,19,25,28,29,33].

Non-randomized comparative studies and propensity score-matched analyses attempted to reduce confounding through comparator groups or statistical adjustment, but residual confounding could not be excluded [12,16,18,24,31,35,36]. Single-arm before–after studies were particularly vulnerable to bias because they lacked a non-SGLT2i comparator group [13,17,20,21,22,24,26,27,30,32,34,37]. In these studies, changes in LV GLS may reflect treatment effects, but may also be influenced by regression to the mean, improved glycemic control, weight loss, blood pressure reduction, background therapy, or time-related clinical changes. Therefore, although the overall direction of evidence favored SGLT2i therapy, the strength of causal inference remains limited.

For this reason, the conclusions should be regarded as hypothesis-generating rather than definitive proof of causality. The strongest causal evidence comes from randomized placebo-controlled or active-comparator designs, but these studies were fewer and produced mixed results. In addition, publication bias or selective reporting of positive strain outcomes cannot be excluded, because formal reporting-bias tests were not appropriate in the absence of meta-analysis and with heterogeneous study designs.

### 4.7. Clinical Implications

The findings of this review suggest that LV GLS may be a useful research endpoint for detecting early myocardial effects of SGLT2i therapy in adults with T2DM, particularly in patients with preserved LVEF, pre-heart failure, or subclinical myocardial dysfunction [13,14,15,22,24,26,32,36,37]. In these settings, conventional echocardiographic parameters may remain unchanged, while strain imaging may identify earlier changes in myocardial performance.

However, the current evidence is not sufficient to recommend routine serial LV GLS assessment solely for monitoring response to SGLT2i therapy in everyday clinical practice. SGLT2 inhibitors are already supported by cardiovascular and cardiorenal outcome evidence, particularly for reducing heart failure-related events and improving outcomes in high-risk patients [1,7,8]. In contrast, LV GLS remains a surrogate imaging marker and should be interpreted as complementary to, rather than a replacement for, established clinical endpoints such as heart failure hospitalization, cardiovascular mortality, renal outcomes, and functional capacity.

Therefore, LV GLS may be most useful in research settings, early disease detection, and phenotyping of subclinical myocardial dysfunction. Future studies should clarify whether SGLT2i-related improvements in LV GLS are associated with meaningful long-term clinical benefits in adults with T2DM.

### 4.8. Strengths and Limitations of This Review

This review has several strengths. It addressed a focused clinical and imaging question, was structured according to a predefined PICO framework, and synthesized original human studies evaluating LV GLS or LV strain in adults with T2DM. The review also considered both randomized and non-randomized evidence, followed systematic review reporting principles, and assessed risk of bias using tools appropriate to study design [48,49].

In addition, LV GLS assessment was not fully standardized across studies, with potential differences in software platforms, acquisition protocols, apical views, frame rates, tracking quality, segmental models, and vendor-specific algorithms. These factors may influence the measured magnitude of GLS and limit the comparability of absolute values across studies.

A further limitation is the narrative rather than quantitative nature of the synthesis. A more quantitative approach could be informative in the future, but only if studies provide comparable numerical data, including baseline and follow-up GLS values, standard deviations, between-group differences, and consistent definitions of meaningful change.

Another limitation is that some populations were highly specific, including patients with HFrEF, HFmrEF, diabetes-related cardiomyopathy, acute coronary syndrome, NAFLD, or breast cancer receiving doxorubicin-based chemotherapy [16,17,18,19,20,21,23,29,31]. These studies are relevant to the broader question of SGLT2i and myocardial deformation, but their findings may not be directly generalizable to all adults with T2DM. Because of this clinical and methodological heterogeneity, quantitative pooling may not be appropriate unless sufficiently comparable numerical data are available.

### 4.9. Future Research

Future studies should include larger randomized controlled trials with standardized speckle-tracking echocardiography protocols, longer follow-up, predefined LV GLS endpoints, and appropriate comparator groups. Consistent reporting of baseline LV GLS, follow-up LV GLS, absolute and percentage change, between-group differences, software platform, segmental model, apical views, frame rate, and acquisition methods would improve comparability across studies. This is particularly important because strain measurements may vary according to acquisition protocol, software, and vendor-specific analysis methods [50].

Further studies should also explore whether treatment effects differ according to baseline GLS impairment, diabetes duration, heart failure phenotype, body mass index, renal function, coronary artery disease, coronary microvascular dysfunction, and background cardiovascular therapy. Priority populations for future randomized trials include adults with T2DM and impaired baseline LV GLS despite preserved LVEF, early diabetic cardiomyopathy, HFpEF or pre-heart failure, stable coronary artery disease or coronary microvascular dysfunction, and asymptomatic high-risk T2DM patients in whom early myocardial dysfunction may still be modifiable.

Future trials should also link GLS changes to clinically meaningful outcomes, including incident heart failure, heart failure hospitalization, exercise capacity, quality of life, cardiovascular events, renal outcomes, and mortality. This would help determine whether LV GLS improvement is only an imaging signal or a surrogate for meaningful clinical benefit.

## 5. Conclusions

This systematic review suggests that SGLT2 inhibitor therapy may be associated with favorable changes in left ventricular global longitudinal strain in adults with type 2 diabetes mellitus, particularly in observational and before–after studies. These findings support the potential role of SGLT2 inhibitors in improving or preserving subclinical left ventricular systolic function, as assessed by strain imaging.

However, the certainty of the evidence remains limited by substantial heterogeneity in study design, patient populations, comparator groups, follow-up duration, SGLT2 inhibitor regimens, and echocardiographic protocols. In addition, many included studies were observational, single-arm, or before–after studies, which limits causal inference. Therefore, although the available evidence is promising, it does not definitively establish that SGLT2 inhibitors improve LV GLS across all adults with type 2 diabetes mellitus.

Further well-designed randomized controlled trials with standardized speckle-tracking echocardiography protocols, predefined LV GLS endpoints, longer follow-up, and clinically relevant outcomes are needed to confirm whether SGLT2 inhibitor-associated changes in LV GLS reflect true myocardial benefit and translate into reduced heart failure progression, cardiovascular events, or mortality.

## Figures and Tables

**Table 1:** Characteristics of the included studies.

Study ID	Country	Population	Sample Size	Intervention (SGLT2i)	Comparator	Main Echocardiographic Outcomes	Primary and Secondary Outcomes	Follow-Up
Gamaza-Chulian 2021 [12]	Spain	Adults with T2DM; outpatient endocrinology setting.	52 (30 SGLT2i, 22 control)	Mixed SGLT2i	Non-SGLT2i therapy	Longitudinal strain, indexed LVM	Improved strain and reduced indexed LVM after 6 months.	6 months
Song 2023 [13]	China	Adults with T2DM newly treated with dapagliflozin; LVEF ≥ 50%.	27 enrolled; 25 completed	Dapagliflozin 10 mg/day	None; before-after	LV GLS, LVEF, EAT thickness	LV GLS improved and EAT thickness decreased after 6 months.	6 months
Lim 2024 [14]	Republic of Korea	Adults with T2DM and pre-heart failure.	102 randomized	Ertugliflozin 5 mg/day	Placebo	LV GLS, LVMI, LVEF, E/e’	Ertugliflozin improved LV GLS, LVMI, and LVEF versus placebo.	24 weeks
Marwick 2025 [15]	Australia	Older adults with T2DM and stage B HF/subclinical LV dysfunction.	139 randomized	Dapagliflozin 10 mg/day	Placebo	LV GLS, LVEF, LVMI, LA function, E/e’	Improved diastolic/LA function and exercise capacity; no significant GLS benefit versus placebo.	6 and 24 months
Oka 2021 [16]	Japan	T2DM-related cardiomyopathy; early vs. advanced disease by CMR.	55 enrolled; 52 completed	Empagliflozin 10 mg/day	No SGLT2i control	LV GLS, E/e’, LVEF, CMR ECV	Empagliflozin improved LV GLS, especially in early DMCMP.	12 months
Tanaka 2020 [17]	Japan	Adults with T2DM and chronic HF.	53	Dapagliflozin 5–10 mg/day	None; before-after	LV GLS, E/e’, LVEF, LAVI	GLS improved after dapagliflozin, especially in HFpEF.	6 months
Lan 2021 [18]	Australia	Adults with T2DM hospitalized for acute coronary syndrome.	44 completed	Empagliflozin 10 or 25 mg/day	No SGLT2i control	LV GLS, LVEF, LVMI, LAVI, E/e’	Reduced LV mass and favorable diastolic changes; no significant between-group GLS change.	3–6 months
Moses 2022 [19]	Canada	T2DM and stable CAD; EMPA-HEART CardioLink-6 substudy.	97 randomized in parent trial	Empagliflozin 10 mg/day	Placebo	CMR LV strain	No significant change in CMR-derived LV peak GLS versus placebo.	6 months
Palmiero/Cesaro 2023 [20]	Italy	T2DM and chronic HFrEF.	31	Mainly empagliflozin 10 mg/day	None; before-after	LVEF, GLS, myocardial work, LA/RV indices	Improved LVEF, GLS, myocardial work, LA function, RV function, and PASP.	6 months
Savcilioglu 2024 [21]	Turkey	HFrEF and T2DM, SGLT2i-naive.	56 patients; 30 healthy controls	Empagliflozin or dapagliflozin	Baseline healthy controls; before-after in patients	LV GLS, LVEF, LA strain, biomarkers	LV GLS and LA strain improved; LVEF remained unchanged.	3 months
Kümet 2026 [22]	Turkey	Adults with T2DM newly started on SGLT2i; stratified by BMI.	614	Empagliflozin or dapagliflozin	None; before-after	LV GLS, LVEF, E/A, e’, E/e’	GLS improved similarly in normal-weight and overweight/obese groups.	6 months
Ozturk 2026 [23]	Turkey	Breast cancer patients with T2DM receiving doxorubicin.	60 (30/30)	Dapagliflozin 10 mg/day	Non-dapagliflozin oral therapy	LVEF, LV GLS, NT-proBNP	Dapagliflozin improved LV GLS and reduced NT-proBNP.	3 months
Shih 2021 [24]	Taiwan	Diabetic patients without symptomatic HF; translational study.	54	Dapagliflozin 10 mg/day	None; before-after	LVEF, diastolic function, LV GLS	Improved strain and diastolic function; experimental data suggested reduced ER stress/fibrosis/apoptosis.	6 months
Eickhoff 2020 [25]	Denmark	T2DM with albuminuria and preserved cardiac function.	36 completed	Dapagliflozin 10 mg/day	Placebo crossover	LVEF, GLS, E/e’, LAVI, LVMI	No significant GLS or LVEF change; minor diastolic composite improvement.	12 weeks per period
Dural 2022 [26]	Turkey	Diabetes without known cardiovascular disease.	62	Empagliflozin	None; before-after	LV GLS, MME efficiency	LV GLS and myocardial mechano-energetic efficiency improved.	3 months
Cortés 2023 [27]	Spain	Diabetes with preserved EF and structural/diastolic abnormalities.	31	Dapagliflozin 10 mg/day	None; before-after	3D LV mass, LV GLS, IVRT, LA/RV strain	Reduced LV mass and improved LV GLS and IVRT.	6–9 months
Nesti 2022 [28]	Italy	T2DM without clinical or echocardiographic heart disease.	56 randomized; 44 completed	Empagliflozin 10 mg/day	Sitagliptin 100 mg/day	LV GLS, strain rates, VO2peak	Neutral overall effects; GLS improved in subgroup with baseline subclinical dysfunction.	1 and 6 months
Attaran 2023 [29]	Iran	T2DM and NAFLD without established ASCVD.	73 (37/36)	Empagliflozin 10 mg/day	Pioglitazone 30 mg/day	GLS, LVEF, diastolic parameters	GLS improved in both groups, without significant between-group difference.	24 weeks
Kuo 2023 [30]	Taiwan	Patients with T2DM receiving canagliflozin.	47	Canagliflozin 100 mg/day	None; before-after	LV GLS, LVEF, LVMI, E/e’, hemodynamics	Favorable changes in LV GLS and hemodynamic/diastolic parameters.	24 weeks
El-Saied 2024 [31]	Egypt	T2DM and stable HFmrEF.	70 (35/35)	Empagliflozin or dapagliflozin	Non-SGLT2i GDMT	LA strain/volumes, LV GLS, LVEF, E/e’	Improved LA function, LV GLS, and diastolic parameters.	6 months
Cheng 2024 [32]	China	Diabetes with normal EF and cardiovascular risk factors.	128	Empagliflozin	None; before-after	LV/RV/LA strain, myocardial work, LVMI	Improved LV GLS, myocardial work, RV strain, and LA strain.	6 months
Lin 2024 [33]	Taiwan	Asymptomatic T2DM with preserved LVEF and no prior SGLT2i use.	76 randomized	Dapagliflozin 10 mg/day add-on	Standard care	GLS, LV dimensions/volumes, LVMI, strain rates	Improved LV remodeling, GLS, and strain rates versus standard care.	24 weeks
Biter 2024 [34]	Turkey	T2DM with preserved EF, with or without CAD.	100	Empagliflozin or dapagliflozin	None; before-after	LVEF, LV strain, remodeling, NT-proBNP	LVEF and strain improved regardless of CAD; NT-proBNP unchanged.	1 and 6 months
Grubić Rotkvić 2024 [35]	Croatia	T2DM and asymptomatic HF stages A/B on metformin-based therapy.	Subgroups from prior cohort	SGLT2i added to metformin	DPP-4 inhibitor	GLS, E/e’, SVI, NT-proBNP, biomarkers	Improved cardiac load and diastolic/systolic function; attenuated sympathetic response.	6 months
Wang 2025 [36]	China	T2DM with preserved LVEF; stratified by diabetes duration.	256 after PSM	Dapagliflozin	Non-SGLT2i therapy	LV GLS, LVEF, LV mass, E/e’, EAT thickness	Better GLS and lower subclinical LV dysfunction rate with SGLT2i, regardless of diabetes duration.	6 months
Karaduman 2025 [37]	Turkey	T2DM with hypertension, preserved LVEF, and antihypertensive treatment.	63	Empagliflozin	None; before-after	GLS, LAVI, LVEF, E/e’, e’, NT-proBNP	GLS and diastolic parameters improved; NT-proBNP, BP, waist, and BMI decreased.	6 months

Abbreviations: CAD, coronary artery disease; CMR, cardiac magnetic resonance; DMCMP, diabetic cardiomyopathy; EAT, epicardial adipose tissue; EF, ejection fraction; GLS, global longitudinal strain; HF, heart failure; HFmrEF, heart failure with mildly reduced ejection fraction; HFpEF, heart failure with preserved ejection fraction; HFrEF, heart failure with reduced ejection fraction; LA, left atrial; LAVI, left atrial volume index; LV, left ventricular; LVEF, left ventricular ejection fraction; LVMI, left ventricular mass index; NT-proBNP, N-terminal pro-B-type natriuretic peptide; PSM, propensity score matching; RV, right ventricular; SGLT2i, sodium-glucose cotransporter-2 inhibitor; T2DM, type 2 diabetes mellitus.

## Data Availability

The original contributions presented in this study are included in the article/Supplementary Material. Further inquiries can be directed to the corresponding author.

## Supplementary Materials

The following supporting information can be downloaded at: https://www.mdpi.com/article/10.3390/jcm15135137/s1, Supplementary File S1: Full Search Strategies; Supplementary File S2: Excluded Studies; Supplementary File S3: Risk of Bias Assessment of Included Studies; Supplementary File S4: PRISMA_2020_Checklist_SGLT2: Completed PRISMA 2020 checklist.

## Abbreviations

The following abbreviations are used in this manuscript:

A′	Late Diastolic Mitral Annular Velocity
ACE2	Angiotensin-Converting Enzyme 2
AF	Atrial Fibrillation
ASCVD	Atherosclerotic Cardiovascular Disease
BMI	Body Mass Index
BNP	B-type Natriuretic Peptide
CAD	Coronary Artery Disease
CKD	Chronic Kidney Disease
CMR	Cardiac Magnetic Resonance
DBP	Diastolic Blood Pressure
DMCMP	Diabetic Cardiomyopathy
DPP-4	Dipeptidyl Peptidase-4
E/A	Ratio of Early to Late Mitral Inflow Velocity
E/e′	Ratio of Early Mitral Inflow Velocity to Early Diastolic Mitral Annular Velocity
E′/e′	Early Diastolic Mitral Annular Velocity
EAT	Epicardial Adipose Tissue
EATT	Epicardial Adipose Tissue Thickness
EF	Ejection Fraction
eGFR	Estimated Glomerular Filtration Rate
Ertu-GLS	Ertugliflozin–Global Longitudinal Strain
GLS	Global Longitudinal Strain
HbA1c	Glycated Hemoglobin
HF	Heart Failure
HFmrEF	Heart Failure with Mildly Reduced Ejection Fraction
HFpEF	Heart Failure with Preserved Ejection Fraction
HFrEF	Heart Failure with Reduced Ejection Fraction
LA	Left Atrium/Left Atrial
LAEF	Left Atrial Emptying Fraction
LAVI	Left Atrial Volume Index
LEAVE-DM	Left Ventricular Dysfunction in Diabetes Mellitus
LV	Left Ventricle/Left Ventricular
LV GLS	Left Ventricular Global Longitudinal Strain
LVEDD	Left Ventricular End-Diastolic Diameter
LVEF	Left Ventricular Ejection Fraction
LVESD	Left Ventricular End-Systolic Diameter
LVGLS	Left Ventricular Global Longitudinal Strain
LVM	Left Ventricular Mass
LVMI	Left Ventricular Mass Index
NAFLD	Non-Alcoholic Fatty Liver Disease
NT-proBNP	N-terminal pro-B-type Natriuretic Peptide
RV	Right Ventricle/Right Ventricular
SBP	Systolic Blood Pressure
SGLT2i	Sodium-Glucose Cotransporter-2 Inhibitor(s)
T2DM	Type 2 Diabetes Mellitus
