# Supplementary material for: Effects of Sodium-Glucose Cotransporter-2 Inhibitors on Left Ventricular Global Longitudinal Strain in Adults with Type 2 Diabetes Mellitus: A Systematic Review
Source: J Clin Med. 2026 Jul 1;15(13):5137. doi: 10.3390/jcm15135137 (PMC13363150; doi:10.3390/jcm15135137)
Supplement: Supplementary file 1 [file jcm-15-05137-s001.zip › Supplementary File S1 Search_Strategy.pdf]

Effects of Sodium-Glucose Cotransporter-2 Inhibitors on Left Ventricular Global Longitudinal Strain in Adults with Type 2 Diabetes Mellitus: A Systematic Review

Supplementary File 1. Full Search Strategies.

The search process was conducted using four electronic databases: PubMed, Scopus, Web of Science, and the Cochrane Library. The strategy was designed to identify original human clinical studies evaluating the effect of sodium-glucose cotransporter-2 inhibitors on left ventricular global longitudinal strain or LV strain in adults with type 2 diabetes mellitus. The search combined controlled vocabulary, where available, with free-text keywords related to type 2 diabetes mellitus, SGLT2 inhibitors, individual SGLT2 inhibitor agents, left ventricular global longitudinal strain, myocardial strain, and speckle-tracking echocardiography. Full reproducible search strategies for each database are provided below. The last search was performed on 01 June 2026.

1. Search Strategy Development

The search strategy was developed according to the PICO framework used in the systematic review. The population component included adults with type 2 diabetes mellitus; the intervention component included SGLT2 inhibitors and individual agents such as empagliflozin, dapagliflozin, canagliflozin, and ertugliflozin; and the outcome component included LV GLS, left ventricular strain, myocardial strain, and strain echocardiography.

In PubMed, Medical Subject Headings (MeSH) were combined with free-text terms. In Scopus, the search was conducted in title, abstract, and keyword fields using the TITLE-ABS-KEY function. In Web of Science, topic-based free-text terms were combined using Boolean operators. In the Cochrane Library, equivalent keyword combinations were used, including proximity operators where applicable.

The search was limited to studies published in English from January 2015 onwards. In addition to database searching, the reference lists of included studies and relevant reviews were manually screened to identify additional eligible articles.

The exact PubMed search syntax was:

(("Diabetes Mellitus, Type 2"[MeSH] OR "type 2 diabetes" OR "type 2 diabetes mellitus" OR T2DM OR T2D)
AND
("Sodium-Glucose Transporter 2 Inhibitors"[MeSH] OR "SGLT2 inhibitor*" OR "SGLT-2 inhibitor*" OR SGLT2i OR SGLT2 OR empagliflozin OR dapagliflozin OR canagliflozin OR ertugliflozin)
AND
("left ventricular global longitudinal strain" OR "global longitudinal strain" OR "LV GLS" OR LVGLS OR GLS OR "left ventricular strain" OR "myocardial strain" OR "speckle-tracking echocardiography" OR "speckle tracking echocardiography" OR "strain echocardiography"))

2. Full Database Search Strategies

Table S1 presents the number of records retrieved from each database and the exact search queries used for the systematic review.

Search Engine	Number of	Exact Search Query

	**Records Found**	
**Pubmed**	48	(("Diabetes Mellitus, Type 2"[MeSH] OR "type 2 diabetes" OR "type 2 diabetes mellitus" OR T2DM OR T2D)<br>AND<br>("Sodium-Glucose Transporter 2 Inhibitors"[MeSH] OR "SGLT2 inhibitor*" OR "SGLT-2 inhibitor*" OR SGLT2i OR SGLT2 OR empagliflozin OR dapagliflozin OR canagliflozin OR ertugliflozin)<br>AND<br>("left ventricular global longitudinal strain" OR "global longitudinal strain" OR "LV GLS" OR LVGLS OR GLS OR "left ventricular strain" OR "myocardial strain" OR "speckle-tracking echocardiography" OR "speckle tracking echocardiography" OR "strain echocardiography"))
**Scopus**	54	TITLE-ABS-KEY (<br>("type 2 diabetes" OR "type 2 diabetes mellitus" OR T2DM OR T2D)<br>AND<br>("SGLT2 inhibitor*" OR "SGLT-2 inhibitor*" OR SGLT2i OR SGLT2 OR empagliflozin OR dapagliflozin OR canagliflozin OR ertugliflozin)<br>AND<br>("left ventricular global longitudinal strain" OR "global longitudinal strain" OR "LV GLS" OR LVGLS OR GLS OR "left ventricular strain" OR "myocardial strain" OR "speckle-tracking echocardiography" OR "speckle tracking echocardiography" OR "strain echocardiography")<br>)
**Web of Science**	129	("type 2 diabetes" OR "type 2 diabetes mellitus" OR T2DM OR T2D) AND ("SGLT2 inhibitor*" OR "SGLT-2 inhibitor*" OR SGLT2i OR SGLT2 OR empagliflozin OR dapagliflozin OR canagliflozin OR ertugliflozin) AND ("left ventricular global longitudinal strain" OR "global longitudinal strain" OR "LV GLS" OR LVGLS OR GLS OR "left ventricular strain" OR "myocardial strain" OR "speckle-tracking echocardiography" OR "speckle tracking echocardiography" OR "strain echocardiography")
**Cochrane library**	69	("type 2 diabetes" OR "type 2 diabetes mellitus" OR T2DM OR T2D) AND ((SGLT2 NEXT inhibitor*) OR (SGLT-2 NEXT inhibitor*) OR SGLT2i OR SGLT2 OR empagliflozin OR dapagliflozin OR canagliflozin OR ertugliflozin) AND ("left ventricular global longitudinal strain" OR "global longitudinal strain" OR "LV GLS" OR LVGLS OR GLS OR "left ventricular strain" OR "myocardial strain" OR "speckle tracking echocardiography" OR "strain echocardiography")
