# Supplementary material for: Effects of Sodium-Glucose Cotransporter-2 Inhibitors on Left Ventricular Global Longitudinal Strain in Adults with Type 2 Diabetes Mellitus: A Systematic Review
Source: J Clin Med. 2026 Jul 1;15(13):5137. doi: 10.3390/jcm15135137 (PMC13363150; doi:10.3390/jcm15135137)
Supplement: Supplementary file 1 [file jcm-15-05137-s001.zip › Supplementary File S2 Excluded_Articles.pdf]

Effects of Sodium-Glucose Cotransporter-2 Inhibitors on Left Ventricular Global Longitudinal Strain in Adults with Type 2 Diabetes Mellitus: A Systematic Review

Supplementar File S2. Excluded Studies

Table S2. Excluded Studies and Reasons for Exclusion

Pavlidis 2026 [37]	The study was excluded because it compares empagliflozin with dulaglutide, a GLP-1 receptor agonist. Although it includes an SGLT2 inhibitor arm and evaluates LV GLS, the intervention is not focused exclusively on the effect of an SGLT2 inhibitor versus placebo, usual care, or control.
Ikonomidis 2025 [40]	The study was excluded because it evaluates GLP-1 receptor agonists, SGLT2 inhibitors, and their combination, rather than focusing exclusively on the independent effect of an SGLT2 inhibitor versus placebo, usual care, or control. Although it includes an empagliflozin arm and cardiac outcomes, the intervention framework includes GLP-1RA and combination therapy.
Korakas 2024 [38]	The study was excluded because the intervention was combined dulaglutide and dapagliflozin compared with DPP-4 inhibitors. Therefore, the independent effect of the SGLT2 inhibitor cannot be isolated. Although the study includes patients with T2DM and evaluates GLS, it does not assess SGLT2 inhibitor therapy alone versus placebo, usual care, or control.
Katogiannis 2024 [39]	The study was excluded because it evaluates liraglutide, empagliflozin, and their combination, rather than focusing exclusively on the independent effect of an SGLT2 inhibitor versus placebo, usual care, or control. Although it includes an empagliflozin arm and evaluates cardiac strain parameters, the intervention framework includes GLP-1RA and combination therapy.
Ikonomidis 2020 [41]	The study was excluded because it evaluates insulin, GLP-1 receptor agonist, SGLT2 inhibitor, and GLP-1RA + SGLT2i combination therapy rather than focusing exclusively on the independent effect of an SGLT2 inhibitor versus placebo, usual care, or control. Although it includes an empagliflozin arm and evaluates GLS/myocardial work outcomes, the intervention framework includes GLP-1RA and combination therapy.
