# Supplementary material for: Effects of Sodium-Glucose Cotransporter-2 Inhibitors on Left Ventricular Global Longitudinal Strain in Adults with Type 2 Diabetes Mellitus: A Systematic Review
Source: J Clin Med. 2026 Jul 1;15(13):5137. doi: 10.3390/jcm15135137 (PMC13363150; doi:10.3390/jcm15135137)
Supplement: Supplementary file 1 [file jcm-15-05137-s001.zip › Supplementary File S3 RoB.pdf]

Effects of Sodium-Glucose Cotransporter-2 Inhibitors on Left Ventricular Global Longitudinal Strain in Adults with Type 2 Diabetes Mellitus: A Systematic Review

Supplementary File S3. Risk of Bias Assessment of Included Studies.

Assessment method

Risk of bias was assessed according to study design. Randomized controlled trials and randomized crossover studies were evaluated using the Cochrane Risk of Bias 2 (RoB 2) tool, while non-randomized studies were evaluated using the Risk Of Bias In Non-randomized Studies of Interventions (ROBINS-I) tool. Assessments were performed independently by two reviewers and finalized by consensus. The effect of interest was the effect of SGLT2 inhibitor therapy on left ventricular global longitudinal strain (LV GLS) or LV strain in adults with type 2 diabetes mellitus, rather than the effect of perfect adherence to treatment. Judgements were made according to the standard domains of each tool and were categorized using the recommended judgement levels for RoB 2 and ROBINS-I. Overall risk of bias was determined according to the domain-level judgements and the guidance of the corresponding assessment tool.

Operationalization of risk of bias assessment in this review

**Target trial / target comparison**	Adults with type 2 diabetes mellitus receiving an SGLT2 inhibitor compared with placebo, standard care, no SGLT2 inhibitor treatment, or another eligible antihyperglycemic therapy, with assessment of LV GLS or LV strain using speckle-tracking echocardiography or an equivalent strain imaging method.
**Unit of assessment**	Study outcome level. The primary outcome assessed for risk of bias was LV GLS or LV strain, as reported in each included study.
**Effect of interest**	The effect of assignment to, initiation of, or treatment with an SGLT2 inhibitor on LV GLS or LV strain, rather than the effect of perfect adherence to therapy.
**Prespecified confounders and co-interventions**	Baseline cardiovascular status; severity and duration of type 2 diabetes mellitus; baseline LV GLS or LV strain; left ventricular ejection fraction; presence of heart failure, coronary artery disease, hypertension, chronic kidney disease, obesity, non-alcoholic fatty liver disease, or other relevant comorbidities; baseline glycemic control; background antihyperglycemic therapy; heart failure guideline-directed medical therapy; antihypertensive, lipid-lowering, or cardioprotective therapy; changes in body weight, blood pressure, HbA1c, renal function, and volume status; follow-up duration; and differences in echocardiographic acquisition, software, and strain analysis protocols.

**Interpretation rule**	Results judged at high, serious, or critical risk of bias were considered limited for causal inference. In this review, confounding, absence of a comparator group, selection of participants, deviations from intended interventions, and heterogeneity in imaging protocols were anticipated to be the dominant sources of bias, particularly because many included studies used observational or before-after designs without a randomized placebo-controlled comparator.

ROBINS-I assessment for non-randomized studies

Study	Outcome assessed	D1	D2	D3	D4	D5	D6	D7	Overall	Support for final judgement
Gamaza-Chulián et al. [11]	LV GLS	Moderate	Moderate	Low	Moderate	Low	Low	Moderate	Moderate	Prospective controlled observational study; non-randomized allocation and residual confounding remain possible.
Song et al. [12]	LV GLS	Serious	Moderate	Low	Moderate	Moderate	Low	Moderate	Serious	Single-arm before-after design without a non-SGLT2i control; changes may reflect time effects, metabolic improvement, or regression to the mean.
Oka et al. [15]	LV GLS	Moderate	Moderate	Low	Moderate	Moderate	Low	Moderate	Moderate	Prospective controlled observational study; non-randomized treatment and disease-stage differences may confound the effect.
Tanaka et al. [16]	LV GLS	Serious	Moderate	Low	Moderate	Moderate	Low	Moderate	Serious	Single-arm before-after study in chronic heart failure; absence of a control group limits causal attribution.
Lan et al. [17]	LV GLS	Moderate	Moderate	Low	Moderate	Low	Low	Moderate	Moderate	Non-randomized controlled study after acute coronary syndrome; selection and treatment indication may have influenced outcomes.
Palmiero/Cesaro et al. [19]	LV GLS	Serious	Moderate	Low	Moderate	Moderate	Low	Moderate	Serious	Small pilot before-after study in HFrEF without a separate clinical control group.
Savcılıoglu et al. [20]	LV GLS	Serious	Moderate	Low	Moderate	Moderate	Low	Moderate	Serious	Prospective follow-up study; healthy controls were not a treatment-effect comparator, and no non-SGLT2i patient control was included.
Kümet et al. [21]	LV GLS	Serious	Moderate	Low	Moderate	Low	Low	Moderate	Serious	Large before-after cohort stratified by BMI but lacking a non-SGLT2i comparator; residual confounding remains possible.
Ozturk et al. [22]	LV GLS	Moderate	Moderate	Low	Moderate	Moderate	Low	Moderate	Moderate	Non-randomized comparative cardio-oncology study; chemotherapy exposure and baseline treatment may confound the effect.
Shih et al. [23]	LV GLS	Serious	Moderate	Low	Moderate	Moderate	Low	Moderate	Serious	Human before-after study without a clinical control group; experimental data support plausibility but not causal inference.
Dural et al. [25]	LV GLS	Serious	Moderate	Low	Moderate	Moderate	Low	Moderate	Serious	Before-after study without a comparator; improvements may reflect metabolic or background-treatment changes.
Cortés et al. [26]	LV GLS	Serious	Moderate	Low	Moderate	Moderate	Low	Moderate	Serious	Single-centre before-after observational study without a control group.
Kuo et al. [29]	LV GLS	Serious	Moderate	Low	Moderate	Moderate	Low	Moderate	Serious	Before-after canagliflozin study without a separate control group; time-related effects may influence results.
El-Saied et al. [30]	LV GLS	Moderate	Moderate	Low	Moderate	Low	Low	Moderate	Moderate	Controlled non-randomized HFmrEF study; absence of randomization leaves potential confounding by baseline therapy and clinical status.
Cheng et al. [31]	LV GLS	Serious	Moderate	Low	Moderate	Moderate	Low	Moderate	Serious	Before-after empagliflozin study without a comparator; strain and myocardial work changes cannot be attributed solely to treatment.

Biter et al. [33]	LV GLS	Serious	Moderate	Low	Moderate	Moderate	Low	Moderate	Serious	Follow-up study without a non-SGLT2i control; CAD status, background therapy, and metabolic changes may confound findings.
Grubić Rotkvić et al. [34]	LV GLS	Moderate	Moderate	Low	Moderate	Low	Low	Moderate	Moderate	Secondary controlled analysis comparing SGLT2i with DPP-4i therapy; residual confounding cannot be excluded.
Wang et al. [35]	LV GLS	Moderate	Moderate	Low	Moderate	Low	Low	Moderate	Moderate	Propensity score matching reduced measured confounding, but unmeasured residual confounding remains possible.
Karaduman et al. [36]	LV GLS	Serious	Moderate	Low	Moderate	Moderate	Low	Moderate	Serious	Retrospective single-centre before-after study without a control group; concurrent therapies were not fully controlled.

Abbreviations: D1, bias due to confounding; D2, bias due to selection of participants; D3, bias in classification of interventions; D4, bias due to deviations from intended interventions; D5, bias due to missing data; D6, bias in measurement of outcomes; D7, bias in selection of the reported result; LV GLS, left ventricular global longitudinal strain; SGLT2i, sodium-glucose cotransporter-2 inhibitor.

RoB 2 assessment for randomized studies

Study	Outcome assessed	D1	D2	D3	D4	D5	Overall	Support for final judgement
Lim et al. [13]	LV GLS	Low	Low	Low	Low	Low	Low risk of bias	Randomized, double-blind, placebo-controlled Ertu-GLS trial with objective STE-based LV GLS assessment.
Marwick et al. [14]	LV GLS	Low	Low	Some concerns	Low	Low	Some concerns	Randomized placebo-controlled LEAVE-DM trial; concerns relate mainly to selection complexity and multiple follow-up assessments.
Moses et al. [18]	CMR LV strain	Low	Low	Some concerns	Low	Low	Some concerns	Randomized placebo-controlled strain substudy; substudy design and limited strain sample introduce some concerns.
Eickhoff et al. [24]	LV GLS	Low	Low	Some concerns	Low	Low	Some concerns	Double-blind randomized crossover trial; small sample and crossover design introduce some concerns.
Nesti et al. [27]	LV GLS	Low	Some concerns	Low	Low	Low	Some concerns	Randomized active-comparator trial; active comparator and subgroup interpretation introduce some concerns.
Attaran et al. [28]	LV GLS	Some concerns	Some concerns	Low	Some concerns	Low	Some concerns	Randomized single-blind active-comparator trial; single blinding and active comparator affect interpretation.
Lin et al. [32]	LV GLS	Low	Some concerns	Low	Low	Low	Some concerns	Randomized controlled add-on therapy study; pragmatic treatment context may introduce some concerns.

Abbreviations: D1, bias arising from the randomization process; D2, bias due to deviations from intended interventions; D3, bias due to missing outcome data; D4, bias in measurement of the outcome; D5, bias in selection of the reported result; LV GLS, left ventricular global longitudinal strain; STE, speckle-tracking echocardiography; CMR, cardiac magnetic resonance
